# HLA-G and HLA-F protein isoform expression in breast cancer patients receiving neoadjuvant treatment

**DOI:** 10.1038/s41598-020-72837-3

**Published:** 2020-09-25

**Authors:** Franziska M. Wuerfel, Hanna Huebner, Lothar Häberle, Paul Gass, Alexander Hein, Sebastian M. Jud, Carolin C. Hack, Marius Wunderle, Rüdiger Schulz-Wendtland, Ramona Erber, Arndt Hartmann, Arif B. Ekici, Matthias W. Beckmann, Peter A. Fasching, Matthias Ruebner

**Affiliations:** 1 Department of Gynaecology and Obstetrics, Erlangen University Hospital, Comprehensive Cancer Center Erlangen-EMN, Friedrich Alexander University of Erlangen–Nuremberg (FAU), Universitätsstrasse 21–23, 91054 Erlangen, Germany; 2 Institute of Diagnostic Radiology, Erlangen University Hospital, Comprehensive Cancer Center Erlangen-EMN, Friedrich Alexander University of Erlangen–Nuremberg (FAU), Erlangen, Germany; 3 Institute of Pathology, Erlangen University Hospital, Comprehensive Cancer Center Erlangen-EMN, Friedrich Alexander University of Erlangen–Nuremberg (FAU), Erlangen, Germany; 4 Institute of Human Genetics, Friedrich Alexander University of Erlangen–Nuremberg (FAU), Erlangen, Germany

**Keywords:** Predictive markers, Breast cancer

## Abstract

The immunosuppressive human leukocyte antigens HLA-G and HLA-F are expressed on trophoblast and malignant cells. Four membrane-bound and three soluble HLA-G protein isoforms have been described, which have different immunosuppressive potentials. HLA-F has three transcript variants, resulting in three different protein isoforms. The aim of this study was to evaluate the prognostic and predictive value of HLA-G and HLA-F protein isoform expression patterns in patients with breast cancer. Core biopsies were taken at diagnosis in patients with HER2+ (n = 28), luminal B-like (n = 49) and triple-negative (n = 38) breast cancers who received neoadjuvant chemotherapy. Expression levels of HLA-F and -G were correlated with the pathological complete response (pCR). Protein expression was determined by Western blot analysis, using two antibodies for each HLA, specific for different isoforms. The protein expression of HLA isoforms did not significantly differ between breast cancer subtypes. However, some initial indications were found for an association between the soluble HLA-G6 protein isoform and pCR in HER2+ breast cancer. The study provides preliminary evidence for the evaluation of HLA-G isoform expression, in particular HLA-G6, as a possible new marker for pCR in HER2+ breast cancer.

## Introduction

The immune system plays an important role in many cancers. In breast cancer (BC), the importance of the immune system has been established in several clinical scenarios. For example, first-line triple-negative advanced breast cancer patients treated with the programmed cell death-ligand 1 (PD-L1) antibody atezolizumab had an improved overall survival when tumour-infiltrating lymphocytes (TILs) showed PD-L1 expression^1^. The number of TILs has also been shown to be an independent predictor of the response to neoadjuvant chemotherapy (NACT)^2–4^. NACT was initially administered in patients with inoperable breast cancer in order to reduce tumour size. Patients who responded well to NACT, as indicated by a decrease in tumour size, had improved overall (OS) and disease-free survival rates^5–7^. The administration of NACT, in patients with operable breast cancer as well, became a standard treatment in view of higher rates of breast-conserving surgery and the opportunity it provides to monitor treatment success in vivo. Treatment success with NACT can range from a minimal reduction in tumour size to a total absence of tumour tissue. The total absence of cancer cells, described as a pathological complete response (pCR), is an important marker for improved overall survival^8^.

It is therefore of particular importance to understand the mechanisms underlying the response to NACT in order to evaluate valid response markers. The mechanisms behind the immune system’s contribution to the outcome of treatment are currently under investigation. The present study focuses on the role of human leukocyte antigens G and F (HLA-G and HLA-F) in this context.

The HLA-G protein is a non-classical major histocompatibility antigen, mainly expressed on extravillous trophoblasts of the placenta and various types of carcinoma, such as lung cancer, gastric cancer and prostate cancer^9–11^. HLA-G predominantly mediates immune evasion and immune suppression by inhibiting cells of the adaptive and innate immune system—e.g., natural killer cells (NK cells), T lymphocytes and B lymphocytes interacting with inhibitory receptors such as leukocyte immunoglobulin-like receptors B1 and B2 (LILRB1 and LILRB2) or killer cell immunoglobulin-like receptor 2DL4 (KIR2DL4)^12,13^. HLA-G can be expressed as four alternatively spliced membrane-bound isoforms (HLA-G1 to HLA-G4). Additionally, in contrast to classical HLA class I genes, HLA-G can also occur in the form of three soluble HLA-G isoforms (HLA-G5 to HLA-G7)^14^. HLA-G1 and the soluble HLA-G5 represent the complete extracellular protein structure, which is composed of three alpha domains (α_1_–α_3_) connected with β_2_ microglobulin (β2M)^15^. The other isoforms are not associated with β2M and differ in their extracellular protein structure. The soluble isoforms show a high degree of structural similarity with the membrane-bound isoforms, but preserve intron 4 (HLA-G5 and -G6) or intron 2 (HLA-G7), leading to a loss of the transmembrane domain^16–18^. It is hypothesized that these seven protein isoforms have distinct immunosuppressive potentials. It is known that both full-length isoforms (HLA-G1 and HLA-G5) interact with the HLA-G-specific receptor via the α_1_ or α_3_ domain^19^. The α_1_ domain binds to the KIR2DL4 receptor, whereas the α_3_ domain is recognized by LILRB1, LILRB2 and CD8^8,20^. Isoforms that lack the α_3_ domain, such as HLA-G3, -G4 and -G7, do not interact with these receptors.

HLA-F is the second, less well known member of the non-classical HLA class I family. The protein structure of HLA-F is similar to that of HLA-G and consists of a leader peptide encoded by exon 1, three alpha domains encoded by exons 2, 3 and 4, a transmembrane domain (exon 5) and a cytoplasmic tail, encoded by exons 6 and 7^21^. In comparison with other HLA genes, the cytoplasmic tail is shorter and varies in length between the three isoforms, which are encoded by three mRNA transcript variants^22^. HLA-F is mainly restricted to the intracellular parts of the cell, but can also be expressed on the cell surface of activated B cells and extravillous trophoblasts^21,23^. It has been suggested that cell surface expression depends on the transport of HLA-F protein to the cell membrane, mediated by its cytoplasmic tail^24^. Similar to HLA-G, HLA-F also interacts with inhibitory receptors LILRB1 and -B2, as well as the activating KIR receptor KIR3DS1^25–27^.

Due to the different immunosuppressive effects of the HLA-G isoforms and the diverse role of HLA-F, these proteins could be of interest for the investigation of pathogenesis and progression and as a therapeutic target for breast cancer. The primary aim of the study was therefore to analyse the distribution of HLA-G and HLA-F across the molecular subgroups of breast cancer. Investigating the association between HLA-G and HLA-F expression and pathological complete response (pCR) after NACT was an additional exploratory aim of the study.

## Methods

### Patient selection

Patients were recruited within the iMODE-B study (Imaging and Molecular Detection of Breast Cancer)^3,28^. Patients were eligible for inclusion if they had an indication for a diagnostic biopsy due to a suspicious breast lesion. The main aim of the iMODE-B study is to identify molecular markers at the time of the first breast cancer diagnosis that are predictive of the prognosis and treatment response.

**Figure 1:**
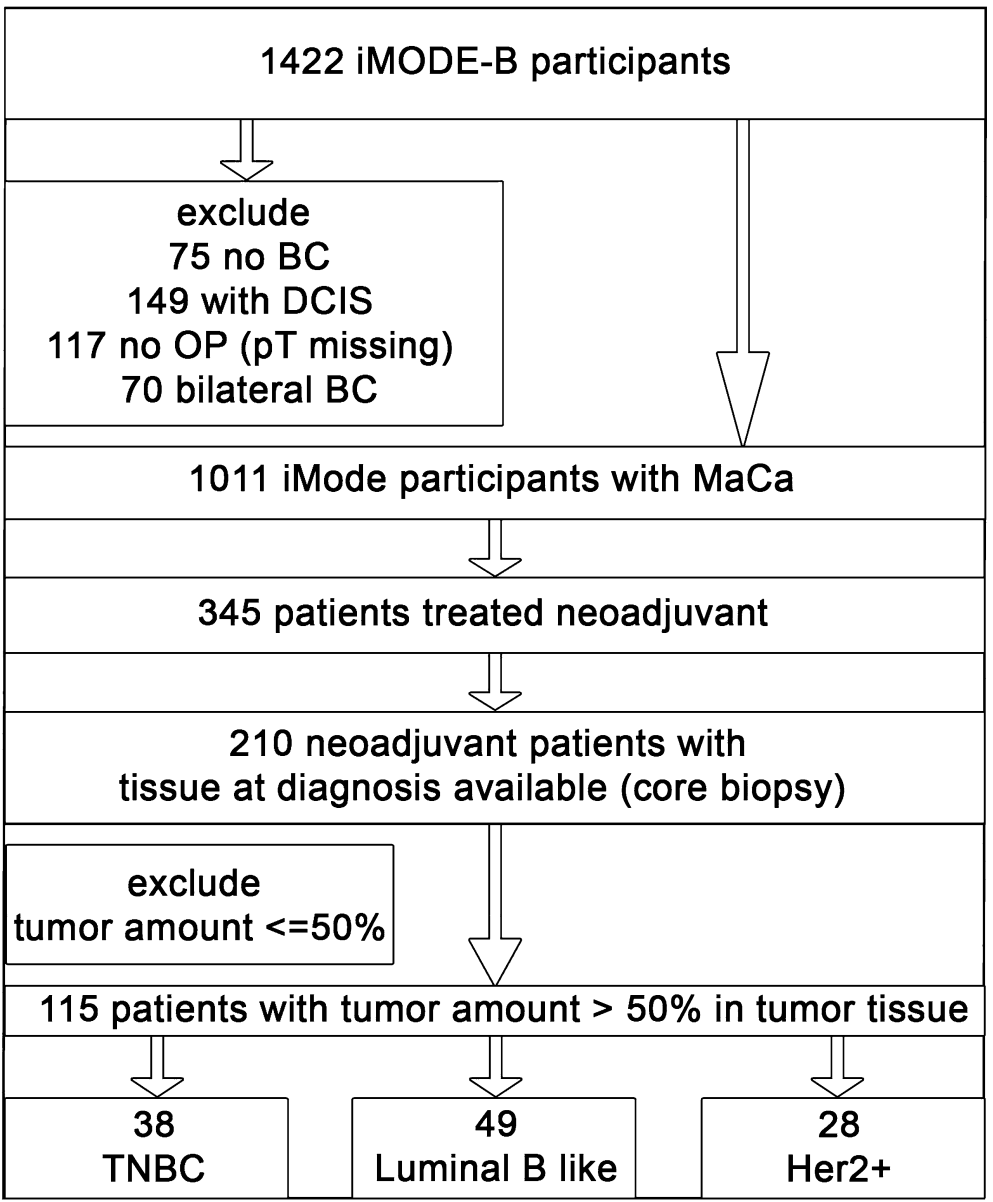
Flow chart showing the patient selection criteria used in the iMODE-B study (Imaging and Molecular Detection of Breast Cancer), with molecular markers at the time of breast cancer diagnosis or progression, molecular detection and imaging detection of breast cancer. *BC* breast cancer, *DCIS* ductal carcinoma in situ, *HER2+* human epidermal growth factor receptor 2-positive, *MaCa* breast cancer, *OP* surgical operation, *pT* pathological staging, *TNBC* triple-negative breast cancer.

A total of 1422 participants were recruited into the iMODE-B study from 2007 to 2017. Patients were excluded in the following hierarchical order (Fig. 1): 75 were not diagnosed with BC (controls as per iMODE-B protocol); 149 patients were diagnosed with ductal carcinoma in situ (DCIS); clinical staging data were missing for 117 patients; and 70 participants had bilateral breast cancer. The study population of iMODE-B patients who were diagnosed with BC at the primary diagnosis therefore comprised 1011 patients. Of these, 345 patients were treated with NACT, among whom 135 participants were excluded due to missing fresh-frozen tissue from the breast cancer core biopsy. The molecular subtypes were classified as follows, in accordance with the St. Gallen guidelines^29^.

- Luminal B-like: hormone receptor (HR)-positive, human epidermal growth factor receptor 2 (HER2)-negative and grading G3.
- Triple-negative breast cancer (TNBC): HR-negative and HER2-negative.
- HER2-positive: HR-positive/negative [estrogen receptor (ER) and/or progesterone receptor (PR) ≥ 1%] and HER2-positive.

To achieve tumour-specific protein results, patients with core biopsies with tumour amounts ≤ 50% were also excluded from the study cohort. The final study population therefore consisted of 115 patients who were treated with NACT in the iMODE-B study (Fig. 1).

Within this cohort, 38 patients were diagnosed with triple-negative breast cancer, 49 with luminal B-like breast cancer and 28 participants with HER2-positive breast cancer.

Pathological complete response (pCR) to NACT was defined in accordance with the semiquantitative scoring system presented by Sinn et al.^30^.

All of the patients provided written informed consent, and the study was approved by the ethics committee of the Medical Faculty of Friedrich Alexander University of Erlangen–Nuremberg.

### Clinical data

Patient data were collected prospectively, and a web-based database was used for documentation, as described previously^31,32^. Patient and tumour characteristics, detailed treatment data and epidemiological data were documented. Follow-up treatments and disease characteristics were collected for up to 10 years after the primary diagnosis^33^. All histological tumour data, such as tumour size, axillary lymph-node status, grading, ER status, PR status and HER2 status were documented. The data are monitored as part of the review process for certification of the breast cancer center and are audited annually^33^. Data obtained from these collection and auditing processes were used in the analysis presented here.

### Sample collection and preparation

Breast cancer biopsies were fresh-frozen in liquid nitrogen immediately after the core biopsy was taken and stored at − 80 °C until further use. Only fresh-frozen biopsies of patients with tumour proportions of more than 50% in the diagnostic core biopsies were chosen for analysis. One fresh-frozen breast cancer core biopsy from each patient was used for protein extraction.

### Protein extraction and quantification

Protein was extracted from fresh-frozen breast cancer core biopsies using RIPA protein lysis buffer (Sigma Aldrich, Taufkirchen, Germany), consisting of 1 mM Na_3_VO_4_, 1.5 mM NaF, 1 mM phenylmethylsulfonyl fluoride (PMSF) and protein inhibitor cocktail (pepstatin, leupeptin and chymostatin each 10 μg/mL). The tissues were homogenized with protein lysis buffer using the Precellys^®^ 24-tissue homogenizer (Bertin Instruments, Frankfurt am Main, Germany) with reinforced tubes (MK28-R hard tissue grinding kit, 2 mL reinforced tubes with screw cap and skirt; VWR, Darmstadt, Germany) with an interval of three times 30 s at 4500 rpm. Afterwards, the tubes were centrifuged at high speed for 1 min. The supernatant was used for further analysis. Protein concentrations were measured using the EZQ protein quantitation kit (Thermo Fisher, Darmstadt, Germany) in accordance with the manufacturer’s instructions. Egg albumin served as the protein standard, provided by the manufacturer.

### Western blot analysis of HLA-G and HLA-F isoforms

In order to analyse the isoform-specific expression pattern of HLA-G and HLA-F, protein samples (15 µg per lane) were separated using 10% sodium dodecyl sulfate polyacrylamide gel electrophoresis (SDS-PAGE). To determine the different isoforms of HLA-G, two commercially available antibodies were used that recognize the membrane-bound isoforms (monoclonal anti-HLA-G mouse antibody, clone 4H84; Abcam, Cambridge, United Kingdom, 1:100) and soluble isoforms (monoclonal anti-HLA-G mouse antibody, clone 5A6G7; Thermo Fisher, Darmstadt, Germany, 1:100), which have both been used in several studies^34–37^. The corresponding bands for the HLA-G isoforms were detected at 38 kDa, as well their glycosylated and ß_2_-microglobulin-associated forms at around 50 kDa for HLA-G1, which were used for quantification^38,39^; 30 kDa for HLA-G2; 22 kDa for HLA-G3; 49 kDa for HLA-G5 [HLA-G5 (36 kDa), associated with ß_2_-microglobulin (13 kDa)]^40^; and 27 kDa for HLA-G6. For the HLA-F analysis, two different commercially available antibodies were used that recognize the N-terminal end of the HLA-F protein (polyclonal anti-HLA-F rabbit antibody; Aviva Systems Biology, San Diego, California, USA) and the C-terminal end (polyclonal anti-HLA-F rabbit antibody; Sigma Aldrich, Taufkirchen, Germany). The corresponding bands for the HLA-F isoforms were detected at 50 kDa for HLA-F1 and 30 kDa for HLA-F3. An anti-mouse HRP-labeled antibody was used as a secondary antibody (monoclonal anti-mouse goat antibody conjugated with horse radish peroxidase; Sigma Aldrich, Taufkirchen, Germany, 1:4000). Protein bands were quantified using ImageJ. Glyceraldehyde 3-phosphate dehydrogenase (GAPDH) was used as an internal protein control (rabbit anti-GAPDH polyclonal antibody, clone 0411, diluted 1:10,000; Santa Cruz Biotechnology, Heidelberg, Germany), and placental tissue was used as a reference sample for each blot. The isoform expression pattern was determined semiquantitatively by normalizing to GAPDH relative to the reference sample (HLA isoform/GAPDH ratio).

### Statistical analysis

The primary objective was to compare the protein levels of the isoforms HLA-F1, HLA-F3, HLA-G1, HLA-G2, HLA-G3, HLA-G5 and HLA-G6 between molecular subtypes (three subtypes: HER2-positive, luminal B-like and triple-negative). A Kruskal–Wallis test was performed for each of these biomarkers. Median values and interquartile ranges (25th percentile and 75th percentile) are presented. Nonparametric methods were used, as the biomarker data were skewed with many low values. High and low levels of protein expression were determined by median.

A secondary, exploratory objective was to compare patients who achieved a pCR with patients who did not achieve a pCR in relation to the isoforms. A Wilcoxon rank sum test was performed for each biomarker.

All of the tests were two-sided, and a *P* value of < 0.05 was regarded as statistically significant. *P* values were not corrected for multiple testing. Calculations were carried out using the R system for statistical computing (version 3.4.0; R Development Core Team, Vienna, Austria, 2017).

### Ethical approval

Approval for the study was obtained from the ethics committee of the Faculty of Medicine at Friedrich Alexander University of Erlangen–Nuremberg and all of the relevant local ethics committees. All procedures were in accordance with the ethical standards of the institutional and/or national research committee and with the 1964 Helsinki Declaration and its later amendments or comparable ethical standards.

### Informed consent

Written informed consent was obtained from the patients as part of the inclusion criteria before they entered the study.

## Results

### Patient characteristics

The study comprised 115 breast cancer core biopsies from patients who received neoadjuvant treatment for breast cancer, with a mean age at diagnosis of 56.7 years (standard deviation, SD, 13.8 years), 45 of whom (39.1%) achieved pCR. Patient characteristics including tumour and lymph-node stage, tumour grade, ER, PR, molecular subtype and pCR are shown in Table 1.

**Table 1:** Patient and tumour characteristics.

Characteristic	Mean or n	SD or %
Age (years)	56.7	13.8
**Tumour stage**		
cT1	32	27.8
cT2	59	51.3
cT3	11	9.6
cT4	13	11.3
**Lymph-node status**		
cN0	88	77.9
cN+	25	22.1
**Tumour grade**		
G2	5	4.3
G3	110	95.7
**Estrogen receptor**		
Negative	45	39.1
Positive	70	60.9
**Progesterone receptor**		
Negative	69	60.0
Positive	46	40.0
**Molecular subtype**		
HER2-positive	28	24.3
Luminal B-like	49	42.6
Triple-negative	38	33.0
**pCR**		
No	70	60.9
Yes	45	39.1

Mean and standard deviation (SD) are shown for continuous characteristics, frequency (n) and percentage (%) for categorical characteristics.

*cT* clinical staging, *cN* clinical nodal status, *HER2* human epidermal growth factor receptor 2, *G2 and G3* grading, *pCR* pathological complete response.

### Expression pattern of HLA-F and HLA-G isoforms in molecular subtypes

**Figure 2:**
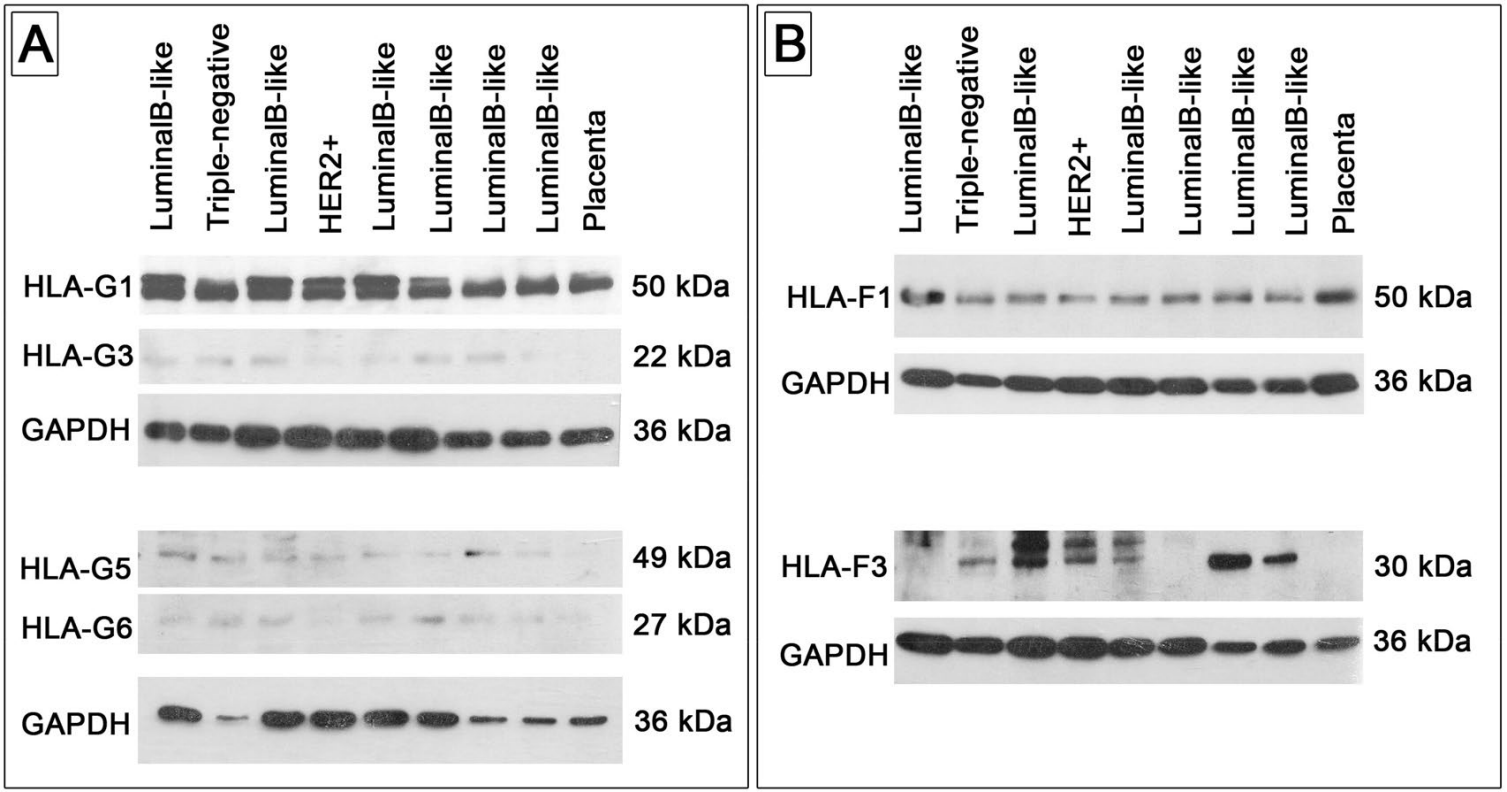
Example Western blots of the HLA antibodies used for expression analysis of HLA-G and HLA-F. (**A**) HLA-G1 and -G3 were assessed using anti-HLA-G antibody clone 4H84 [cropped blots images of HLA-G1 and -G3 as well as GAPDH after stripping are obtained from the same blot; see supplemental figure HLA-G1 (4H84)]. Soluble HLA-G isoforms HLA-G5 and -G6 were assessed using anti-HLA-G antibody clone 5A6G7 [cropped blots images of HLA-G5 and -G6 as well as GAPDH after stripping are obtained from the same blot; see supplemental figure HLA-G (5A6G7)]. (**B**) HLA-F1 expression was measured using an anti-HLA-F antibody Aviva [cropped blots images of HLA-F1 as well as GAPDH after stripping are obtained from the same blot; see supplemental figure HLA-F (Aviva)]. HLA-F3 expression was assessed using an anti-HLA-F antibody from Sigma Aldrich [cropped blots images of HLA-F3 as well as GAPDH after stripping are obtained from the same blot; see supplemental figure HLA-F (Sigma)]. *HER2* human epidermal growth factor receptor 2, *HLA* human leukocyte antigen, *kDa* kilodalton.

The initial aim was to analyse the distribution of the HLA-F and HLA-G isoforms between the molecular subtypes. Western blot analysis was therefore performed with two different antibodies for each HLA, representing different isoforms in relation to their molecular weight. Antibodies were used to detect HLA-F1 and HLA-F3 isoforms, the membrane-bound isoforms HLA-G1, HLA-G2 and HLA-G3, and the soluble isoforms HLA-G5 and HLA-G6 (Table 2). No expression was detected for HLA-G2. In contrast, a high level of expression was noted for HLA-G1 in all subtypes and HLA-G3 in luminal B-like and triple-negative breast cancer core biopsies. However, HLA-F and HLA-G isoform expression did not differ significantly between the molecular subtypes (Table 2 and Fig. 2).

**Table 2:** HLA-G and -F isoform distribution in the molecular subtypes.

Biomarker	HER2+ (n = 28)	Luminal B-like (n = 49)	Triple-negative (n = 38)	*P* value
HLA-F1	0.26 (0.09, 0.46)	0.27 (0.19, 0.44)	0.27 (0.15, 0.5)	0.82
HLA-F3	0.58 (0.10, 2.23)	0.57 (0.15, 2.66)	0.92 (0.26, 2.11)	0.76
HLA-G1	2.92 (1.33, 5.97)	2.85 (1.93, 4.54)	2.69 (1.5, 8.86)	0.80
HLA-G2	0.00 (0.00, 0.00)	0.00 (0.00, 0.00)	0.00 (0.00, 0.00)	0.92
HLA-G3	1.48 (0.74, 3.87)	2.16 (0.74, 3.96)	2.57 (1.37, 4.99)	0.17
HLA-G5	1.87 (0.79, 3.22)	1.37 (0.50, 3.18)	1.17 (0.35, 5.95)	0.72
HLA-G6	0.18 (0.10, 0.45)	0.19 (0.11, 0.47)	0.19 (0.05, 0.37)	0.73

Protein bands from Western blots were quantified using ImageJ. GAPDH was used as an internal protein control, and placental tissue was used as a reference sample for each blot. The isoform expression pattern was determined semiquantitatively by normalizing to GAPDH relative to the reference sample (HLA isoform/GAPDH ratio). The median and interquartile range (25th percentile, 75th percentile) of the HLA isoform/GAPDH ratio were calculated for each molecular subtype, and *P* values were calculated using the Kruskal–Wallis test.

*HER2* human epidermal growth factor receptor 2, *HLA* human leukocyte antigen.

### Association of HLA-F and HLA-G protein isoform expression with pCR status

In addition, HLA-F and HLA-G isoform distribution was analysed in patients with (“pCR”) and without pCR (“no pCR”). However, no differences were observed between these two cohorts for any of the isoforms analysed (Table 3).

**Table 3:** HLA-G and -F isoform distribution in patients with and without a pathological complete response (pCR).

Biomarker	No pCR (n = 70)	pCR (n = 45)	P value
HLA-F1	0.27 (0.15, 0.56)	0.26 (0.15, 0.41)	0.60
HLA-F3	0.50 (0.13, 2.61)	0.86 (0.14, 2.22)	0.78
HLA-G1	2.72 (1.68, 6.35)	2.90 (1.36, 6.37)	0.85
HLA-G2	0.00 (0.00, 0.00)	0.00 (0.00, 0.00)	0.60
HLA-G3	2.16 (0.86, 4.25)	2.42 (0.86, 4.34)	0.81
HLA-G5	1.35 (0.50, 4.92)	1.86 (0.35, 4.15)	0.77
HLA-G6	0.16 (0.08, 0.45)	0.20 (0.10, 0.44)	0.64

Protein bands from Western blots were quantified using ImageJ. GAPDH was used as an internal protein control, and placental tissue was used as a reference sample for each blot. The isoform expression pattern was determined semiquantitatively by normalizing to GAPDH relative to the reference sample (HLA isoform/GAPDH ratio). The median and interquartile range (25th percentile, 75th percentile) of the HLA isoform/GAPDH ratio were calculated for pCR and no pCR, and *P* values were calculated using the Wilcoxon rank sum test.

*HLA* human leukocyte antigen, *pCR* pathological complete response.

For luminal B-like breast cancer samples, an overall low percentage (12–22%) of patients who achieved pCR was observed, independently of the expression of HLA-F and HLA-G isoforms, in comparison with the pCR rates in the HER2+ and triple-negative BC subcohorts (46–86% and 32–71%, respectively) (Table 4). When the molecular subtypes were divided into those with low and high levels of isoform expression relative to the median, it was found that 86% of the patients with HER2+ and HLA-G6 low tumours had a pCR, in comparison with only 46% in the high-level HLA-G6 group (Table 4). In contrast, the triple-negative subtype cohort showed a high pCR rate (62% and 68%, respectively) when HLA-F3 and HLA-G6 expression levels were also defined as “high” (Table 4).

**Table 4:** Descriptive correlation of HLA-G and HLA-F isoforms with low and high levels of expression and pathological complete response status in the subtypes.

	HER2+ (n = 28)		Luminal B-like (n = 49)		Triple-negative (n = 38)	
	No pCR	pCR	No pCR	pCR	No pCR	pCR
Low HLA-F1	5 (36%)	9 (64%)	19 (79%)	5 (21%)	10 (53%)	9 (47%)
High HLA-F1	5 (36%)	9 (64%)	22 (88%)	3 (12%)	9 (47%)	10 (53%)
Low HLA-F3	5 (33%)	10 (67%)	21 (84%)	4 (16%)	11 (65%)	6 (35%)
High HLA-F3	5 (42%)	7 (58%)	20 (83%)	4 (17%)	8 (38%)	13 (62%)
Low HLA-G1	6 (46%)	7 (54%)	19 (86%)	3 (16%)	9 (45%)	11 (55%)
High HLA-G1	4 (29%)	10 (71%)	20 (83%)	4 (17%)	8 (50%)	8 (50%)
Low HLA-G2	7 (32%)	15 (68%)	33 (85%)	6 (15%)	17 (55%)	14 (45%)
High HLA-G2	3 (50%)	3 (50%)	8 (80%)	2 (20%)	2 (29%)	5 (71%)
Low HLA-G3	6 (35%)	11 (65%)	21 (88%)	3 (12%)	7 (47%)	8 (53%)
High HLA-G3	4 (36%)	7 (64%)	18 (78%)	5 (22%)	11 (50%)	11 (50%)
Low HLA-G5	4 (36%)	7 (64%)	20 (83%)	4 (17%)	12 (57%)	9 (43%)
High HLA-G5	6 (35%)	11 (65%)	18 (82%)	4 (18%)	7 (41%)	10 (59%)
Low HLA-G6	2 (14%)	12 (86%)	21 (88%)	3 (12%)	13 (68%)	6 (32%)
High HLA-G6	7 (54%)	6 (46%)	20 (80%)	5 (20%)	6 (32%)	13 (68%)

Protein bands from Western blots were quantified using ImageJ. GAPDH was used as an internal protein control, and placental tissue was used as a reference sample for each blot. The isoform expression pattern was determined semiquantitatively by normalizing to GAPDH relative to the reference sample (HLA isoform/GAPDH ratio). The frequencies of high and low levels of expression were determined using the median of the HLA isoform/GAPDH ratio.

*HLA* human leukocyte antigen, *HER2* human epidermal growth factor receptor 2, *pCR* pathological complete response.

## Discussion

HLA-G is one of the “non-classical” HLA antigens (class Ib) and is thought to have immunosuppressive effects^15^. Isoform-specific differences have not previously been investigated. Large studies on HLA-G expression, as conducted by de Kruijf et al., mostly evaluated using immunohistochemistry, have reported a positive correlation between HLA-G protein expression, shorter disease-free survival and advanced disease stages^34,41^. In the present study, however, correlation analyses between isoform-specific HLA-G and HLA-F expression and overall survival were not yet possible, as the study also included patients with recently diagnosed breast cancer. However, it was found that in the luminal B-like breast cancer subtype, only a small percentage of patients achieved pCR with NACT, independently of the HLA-G and HLA-F subtypes analysed^5^. In the HER2+ subtype, low levels of HLA-G6 expression were associated with a high rate of pCR. The association between HLA-G6 protein expression and the pCR rate was also investigated by König et al. in breast cancer patients^42^. In contrast to the present study, they used an enzyme-linked immunosorbent assay (ELISA) to measure HLA-G5/G6 together in vesicular-derived soluble HLA-G, as well as total soluble HLA-G serum levels in breast cancer patients before and after NACT. In plasma samples from patients with ER+ breast cancer before receiving NACT, they observed a significant correlation between exosome-derived soluble HLA-G levels, tumour progression and overall survival, but not for pCR^42^. Since König et al. measured both soluble HLA-G isoforms (G5/G6) at once, these data are at least partly in concordance with the present results regarding non-significant correlations between HLA-G5/G6 plasma levels and pCR. In order to analyse isoforms, the Western blot technique was used in the present study, by separating the proteins on the basis of their sizes and thus making it possible to distinguish between two or more isoforms with only a single antibody.

With regard to triple-negative BC, relationships between HLA-G and clinical parameters such as low HLA-G6 or HLA-F3 expression and low pCR rates were observed, but these findings did not reach statistical significance due to the small cohort size. These data are not in line with current observations. Dong et al. reported significantly high HLA-G protein expression using immunohistochemistry in non-luminal breast cancer subtypes (HER2+ and TNBC)^43^, whereas He et al. did not observe a significant association between high HLA-G expression and HER2+ status^11^. In the present study of HER2+ breast cancer, showing higher pCR rates with high HLA-G1 expression, however, none of the membrane-bound HLA-G isoforms revealed any significant association with pCR. Several studies on overall HLA-G expression, evaluated using immunohistochemistry, have reported an association between HLA-G protein expression and shorter disease-free survival and advanced disease stage in 41–70% of cases^41^. In addition, de Kruijf et al. found that HLA-G was an independent prognostic marker for reduced overall survival in patients with HLA class I negative breast cancer^34^. These data indicate that membrane-bound HLA-G may be well suited for use as a prognostic marker for overall survival as well as disease-free survival, rather than as a predictor for pCR in breast cancer core biopsies. The reason for this controversial finding might be the use of the anti-HLA-G antibody 4H84, which is known to recognize HLA class Ia molecules to a certain degree as well, and also the small sample size in the different subgroups.

A possible mechanism behind the high level of HLA-G isoform secretion and low pCR rates after NACT is the immunosuppressive potential of this particular HLA-G isoform, which is mediated by its α_1_ and α_3_ domains. As already mentioned, the α_1_ domain inhibits the cytolytic activity of NK cells (CD56^dim^)—via the KIR2DL4 receptor, for instance—whereas the α_3_ domain interacts with the LILRB1 and LILRB2 receptors, inhibiting NK cells, T cells and B cells, as well as dendritic cells and macrophages^8,20^. NACT is able to enhance the general immune response, as is evident from increased immune cell infiltration into the stroma^44,45^. This suggests that soluble isoforms may contribute to an immunosuppressive environment in the proximity of tumours and inhibit the approach of immune cells. In addition, soluble HLA-G isoforms may also act indirectly by inducing HLA-G5-positive or HLA-G6-positive immune cells, which exert immunosuppressive effects. This indirect, secondary mechanism is already well described for the isoforms HLA-G1 and -G5, which are able to induce the generation of HLA-G-positive regulatory T cells (Tregs) and tolerogenic dendritic cells (DC-10), as well as HLA-G-positive macrophages (via trogocytosis)^10,46^. These indirect mechanisms may be responsible for a decreased immune response during and after NACT. This hypothesis is supported by Dong et al., who reported that a high level of HLA-G expression was inversely associated with TIL infiltration in breast cancer^43^.

The impact of HLA-F expression in cancer is a matter of controversy. Harada et al. reported that HLA-F expression is significantly associated with tumour size and a poorer clinical outcome in breast cancer^47^. However, in another study by Zhang et al. on gastric cancer, the authors were unable to show any correlation between HLA-F expression and parameters such as overall survival and tumour stage^48^. In contrast, Ishigami et al. observed a strong correlation with 5-year survival rates and the degree of cancer cell invasion in gastric cancer^49^. The findings of the present study may suggest that there is an impact of expression of HLA-F protein isoforms F1 and F3 and pCR in patients with luminal B-like breast cancer. Expression of HLA-F1 and -F3 was associated with low rates of pCR (12–21%); however, a significant association was not detected due to the small cohort size in luminal B-like cases. These findings are consistent with those of Harada et al., who used immunohistochemistry to measure HLA-F in the tumour node and at the invasive front of the tumour and found that HLA-F was a marker for poorer outcomes in clinical stage II breast cancer^47^. Similar results have been observed in triple-negative breast cancer, where low HLA-G6 and HLA-F3 protein expression was associated with higher pCR rates. These data are partly in line with the observations by Yau et al., in which low mRNA HLA-F expression in combination with other genes served as a poor prognostic marker in TNBC^50^.

However, Western blot analysis has limitations. It is subject to variabilities such as temperature changes and washing during performance^51^. Additionally, film-based detection does not always allow detection of a broad dynamic range. To use Western blotting for quantitative protein detection, it is advisable to determine the linear and quantitative dynamic range of each HLA molecule, and this might generate more significant correlations with pathological clinical data^52^.

An additional limitation of the present study is the limited number of patients, especially for the intrinsic subtypes. The small size of the cohort is also a weakness in relation to the pCR rates obtained in the study. The study shows for the first time that HLA-G6 may be a possible predictive marker for pCR in HER2+ breast cancer. However, this finding needs to be statistically verified in a larger cohort. In addition to verifying the results, enlarging the study cohort could allow adequate statistical analysis to fully determine the correlation between HLA-F and HLA-G isoform expression and pCR.

The exclusion of patients with less than 50% tumour cells in the tissue is a weakness of this study, since a high level of stromal content is associated with a poorer clinical outcome^53^. This subpopulation of patients was excluded—as HLA-G and -F are mainly expressed in tumour, but not in stromal tissue—in order to maximize the tumour tissue content within the lysate. Tumour stromal tissue contains a low number of immune cells^54^, indicating a weak immune response. A poor immune response might be induced by soluble factors such as HLA-G5 or -G6 creating an immunosuppressive environment around the tumour tissue. This was observed by Dong et al., in a study in which high HLA-G expression was associated with low immune cell infiltration^43^.

In summary, this study suggests that HLA-G and HLA-F may play an immunoregulatory role in breast cancer, on the basis of specific analysis of isoforms and splicing variants. Some of the isoforms have different effects on the immune system. It also needs to be taken into consideration that not all of these isoforms are membrane-bound and that some exist as soluble variants, such as HLA-G6. This implies that (breast) cancer cells with high levels of HLA-G6 synthesis not only have a negative impact on TILs, but are also able to act on the surrounding tissue and possibly on the entire immune system.

## References

[1] Schmid P (2018). Atezolizumab and nab-paclitaxel in advanced triple-negative breast cancer. N. Engl. J. Med..

[2] Denkert C (2018). Tumour-infiltrating lymphocytes and prognosis in different subtypes of breast cancer: A pooled analysis of 3771 patients treated with neoadjuvant therapy. Lancet Oncol..

[3] Würfel F (2018). TILGen: A program to investigate immune targets in breast cancer patients—First results on the influence of tumor-infiltrating lymphocytes. Breast Care.

[4] Criscitiello C, Esposito A, Trapani D, Curigliano G (2016). Prognostic and predictive value of tumor infiltrating lymphocytes in early breast cancer. Cancer Treat. Rev..

[5] von Minckwitz G (2012). Definition and impact of pathologic complete response on prognosis after neoadjuvant chemotherapy in various intrinsic breast cancer subtypes. J. Clin. Oncol..

[6] Fasching PA (2011). Ki67, chemotherapy response, and prognosis in breast cancer patients receiving neoadjuvant treatment. BMC Cancer.

[7] Cortazar P (2014). Pathological complete response and long-term clinical benefit in breast cancer: the CTNeoBC pooled analysis. Lancet.

[8] Apps R, Gardner L, Sharkey AM, Holmes N, Moffett A (2007). A homodimeric complex of HLA-G on normal trophoblast cells modulates antigen-presenting cells via LILRB1. Eur. J. Immunol..

[9] King A (1997). Uterine NK cells and trophoblast HLA class I molecules. Am. J. Reprod. Immunol..

[10] Carosella, E. D., Rouas-Freiss, N., Roux, D. T.-L., Moreau, P. & LeMaoult, J. In Chapter Two - HLA-G: An Immune Checkpoint Molecule, *Advances in Immunology* Vol. 127 (ed W. Alt Frederick) 33–144 (Academic Press, 2015). 10.1016/bs.ai.2015.04.001 26073983

[11] He X (2010). HLA-G expression in human breast cancer: Implications for diagnosis and prognosis, and effect on allocytotoxic lymphocyte response after hormone treatment in vitro. Ann. Surg. Oncol..

[12] Young NT (2008). The inhibitory receptor LILRB1 modulates the differentiation and regulatory potential of human dendritic cells. Blood.

[13] Rouas-Freiss N, Moreau P, Menier C, Carosella ED (2003). HLA-G in cancer: a way to turn off the immune system. Semin Cancer Biol.

[14] Ishitani A, Geraghty DE (1992). Alternative splicing of HLA-G transcripts yields proteins with primary structures resembling both class I and class II antigens. Proc. Natl. Acad. Sci. USA.

[15] Carosella ED, Favier B, Rouas-Freiss N, Moreau P, Lemaoult J (2008). Beyond the increasing complexity of the immunomodulatory HLA-G molecule. Blood.

[16] Fujii T, Ishitani A, Geraghty DE (1994). A soluble form of the HLA-G antigen is encoded by a messenger ribonucleic acid containing intron 4. J. Immunol. (Baltimore, Md.: 1950).

[17] Sangrouber D (2007). Cellular co-localization of intron-4 containing mRNA and HLA-G soluble protein in melanoma analyzed by fluorescence in situ hybridization. J. Immunol. Methods.

[18] Paul P (2000). Identification of HLA-G7 as a new splice variant of the HLA-G mRNA and expression of soluble HLA-G5, -G6, and -G7 transcripts in human transfected cells. Hum. Immunol..

[19] Fda SN (2016). Soluble monomers, dimers and HLA-G-expressing extracellular vesicles: The three dimensions of structural complexity to use HLA-G as a clinical biomarker. Hla.

[20] HoWangYin KY (2012). Multimeric structures of HLA-G isoforms function through differential binding to LILRB receptors. Cell. Mol. Life Sci. CMLS.

[21] Kochan G, Escors D, Breckpot K, Guerrero-Setas D (2013). Role of non-classical MHC class I molecules in cancer immunosuppression. OncoImmunology.

[22] Sim MJW, Sun PD (2017). HLA-F: A new kid licensed for peptide presentation. Immunity.

[23] Lee N, Ishitani A, Geraghty DE (2010). HLA-F is a surface marker on activated lymphocytes. Eur. J. Immunol..

[24] Boyle LH, Gillingham AK, Munro S, Trowsdale J (2006). Selective export of HLA-F by its cytoplasmic tail. J. Immunol..

[25] Burian A (2016). HLA-F and MHC-I open conformers bind natural killer cell Ig-like receptor KIR3DS1. PLoS ONE.

[26] Goodridge JP, Burian A, Lee N, Geraghty DE (2013). HLA-F and MHC class I open conformers are ligands for NK cell Ig-like receptors. J. Immunol..

[27] Lepin EJM (2000). Functional characterization of HLA-F and binding of HLA-F tetramers to ILT2 and ILT4 receptors. Eur. J. Immunol..

[28] Erber R (2018). TILGen study-immunological targets in patients with breast cancer: Influence of tumor-infiltrating lymphocytes. Pathologe.

[29] Goldhirsch A (2011). Strategies for subtypes—Dealing with the diversity of breast cancer: highlights of the St. Gallen International Expert Consensus on the Primary Therapy of Early Breast Cancer 2011. Ann. Oncol..

[30] Sinn HP (1994). Histologische Regression des Mammakarzinoms nach primärer (neoadjuvanter) Chemotherapie. Geburtshilfe Frauenheilkd.

[31] Hein A (2016). Computerized patient identification for the EMBRACA clinical trial using real-time data from the PRAEGNANT network for metastatic breast cancer patients. Breast Cancer Res. Treat..

[32] Fasching PA (2015). Biomarkers in patients with metastatic breast cancer and the PRAEGNANT study network. Geburtshilfe Frauenheilkd.

[33] Beckmann MW (2011). Quality assured health care in certified breast centers and improvement of the prognosis of breast cancer patients. Onkologie.

[34] de Kruijf EM (2010). HLA-E and HLA-G expression in classical HLA class I-negative tumors is of prognostic value for clinical outcome of early breast cancer patients. J. Immunol. (Baltimore, Md.: 1950).

[35] Swets M (2016). HLA-G and classical HLA class I expression in primary colorectal cancer and associated liver metastases. Hum. Immunol..

[36] Lefebvre S (2002). Specific activation of the non-classical class I histocompatibility HLA-G antigen and expression of the ILT2 inhibitory receptor in human breast cancer. J. Pathol..

[37] Zhang X (2016). Lesion HLA-G5/-G6 isoforms expression in patients with ovarian cancer. Hum. Immunol..

[38] Kleinberg L (2006). Expression of HLA-G in malignant mesothelioma and clinically aggressive breast carcinoma. Virchows Arch..

[39] Gonzalez Á, Alegre E, Arroyo A, LeMaoult J, Echeveste JI (2011). Identification of circulating nonclassic human leukocyte antigen G (HLA-G)-like molecules in exudates. Clin. Chem..

[40] Favier B (2011). Tolerogenic function of dimeric forms of HLA-G recombinant proteins: A comparative study in vivo. PLoS ONE.

[41] Lin A, Yan W-H (2015). Human leukocyte antigen-G (HLA-G) expression in cancers: Roles in immune evasion, metastasis and target for therapy. Mol. Med..

[42] Konig L (2016). The prognostic impact of soluble and vesicular HLA-G and its relationship to circulating tumor cells in neoadjuvant treated breast cancer patients. Hum. Immunol..

[43] Dong D-D (2012). Importance of HLA-G expression and tumor infiltrating lymphocytes in molecular subtypes of breast cancer. Hum. Immunol..

[44] Pelekanou V (2017). Effect of neoadjuvant chemotherapy on tumor-infiltrating lymphocytes and PD-L1 expression in breast cancer and its clinical significance. Breast Cancer Res. BCR.

[45] Teng F (2015). Tumor infiltrating lymphocytes (TILs) before and after neoadjuvant chemoradiotherapy and its clinical utility for rectal cancer. Am. J. Cancer Res..

[46] LeMaoult J (2007). Immune regulation by pretenders: Cell-to-cell transfers of HLA-G make effector T cells act as regulatory cells. Blood.

[47] Harada A (2015). Clinical implication of human leukocyte antigen (HLA)-F expression in breast cancer. Pathol. Int..

[48] Zhang JG, Zhang X, Lin A, Yan WH (2013). Lesion HLA-F expression is irrelevant to prognosis for patients with gastric cancer. Hum. Immunol..

[49] Ishigami S (2015). Human leukocyte antigen (HLA)-E and HLA-F expression in gastric cancer. Anticancer Res..

[50] Yau C (2013). An optimized five-gene multi-platform predictor of hormone receptor negative and triple negative breast cancer metastatic risk. Breast Cancer Res..

[51] Hirano S (2012). Western blot analysis. Methods Mol. Biol. (Clifton, N.J.).

[52] Taylor SC, Berkelman T, Yadav G, Hammond M (2013). A defined methodology for reliable quantification of western blot data. Mol. Biotechnol..

[53] Roeke T (2017). The prognostic value of the tumour-stroma ratio in primary operable invasive cancer of the breast: A validation study. Breast Cancer Res. Treat..

[54] Tchou J, Conejo-Garcia J (2012). Targeting the tumor stroma as a novel treatment strategy for breast cancer: Shifting from the neoplastic cell-centric to a stroma-centric paradigm. Adv. Pharmacol..

